# Different sizes of position games and official matches in youth professional soccer player

**DOI:** 10.5114/biolsport.2025.144297

**Published:** 2024-10-23

**Authors:** Jose A. Asian-Clemente, Jose Vicente Beltran-Garrido, Bernardo Requena

**Affiliations:** 1 Football Science Institute, FSI lab, Granada, Spain; 2 Department of Sport sciences, Universidad Pablo de Olavide, Seville, Spain; 3 Physical Exercise and Performance Research Group, Department of Education Sciences, School of Humanities and Communication Sciences, Universidad Cardenal Herrera-CEU, CEU Universities, Castellon de la Plana, Spain

**Keywords:** Small-sided games, Time-motion, Football, Tactical, Area per player

## Abstract

The objectives of this study were to compare the external load of position games and matches in soccer players and to assess the effect of different pitch sizes on position games in relation to competition. Twenty-five players (age: 21.9 ± 1.9 years; height: 177.9 ± 5.2 cm; weight: 75.5 ± 4.8 kg) were evaluated during 37 official matches and 18 position games classified according to their pitch sizes: Small, Medium and Large (50.8 ± 6.6 m^2^, 80.5 ± 4.6 m^2^ and 115.9 ± 25.2 m^2^ relative area per player, respectively). Using a GPS system, total distance (DC), DC > 21 km · h^−1^, peak speed, and maximal accelerations and decelerations lower and higher than 3 m · s^−2^ (Acc_<3_; Acc_>3_; Dec_<3_ and Dec_>3_, respectively) were recorded. Mixed model analyses were used to compare the effects of the game condition and of space dimensions on the dependent parameters. During the position games, lower values of DC, DC > 21 km · h^−1^, peak speed, Acc_<3_, Dec_<3_, maximal accelerations and maximal decelerations were reported than in matches. Position games showed higher values than matches for the Acc_>3_ and Dec_>3_. All analysed variables reached statistical significance (all *p* < 0.001). Small, Medium and Large position games significantly achieved lower DC > 21 km · h^−1^, peak speed, Acc_<3_, Dec_<3_, maximal accelerations and maximal decelerations, but significantly higher Acc_>3_ and Dec_>3_ (all *p* < 0.05) than matches. The data showed that position games of 9 vs. 9 + 2 with a relative area per player ≤ 115.9 m^2^ present a different external load than matches.

## INTRODUCTION

The growing interest of coaching staff, coupled with the evolution of time-motion systems, has led to a substantial increase in knowledge related to the running demands of soccer matches in recent years [1]. One of the objectives of monitoring is to find an optimal training stimulus to prescribe the volume and intensity of exercises [2], ensuring that athletes receive a suitable training load [3]. This monitoring enables the practitioner to assess whether athletes have successfully executed the planned training and how well they have managed physical stress [4]. Some authors argue that, to avoid underestimating or overestimating players’ physical stress, contextualising the stimulus is an essential aspect [5]. In general, it can be affirmed that the best way to contextualise the stimulus is to mimic competition [6]. It has been established that the more specific or representative the training tasks, the more transferable are the players’ adaptations from training to competition [7] and greater physical and physiological improvements can be achieved [8]. Training tasks must be chosen based on the expectations of players in official games [7], and therefore coaches must develop tailored training programs that incorporate drills that simulate the specific demands of the game to effectively prepare soccer players [9]. Due to their similarity to actual competition, small-sided games are preferred by most soccer coaches over other drills [10] and have become the most frequently used tasks for this purpose [11]. Small-sided games are modified games played on reduced pitch areas using adapted rules and involving smaller numbers of players compared to the official match play [12]. However, there is a scarcity of studies that have conducted a comparison between running requirements in competitive matches and small-sided games, with the goal of achieving more tailored adaptations and enhancing the performance of soccer players [13].

Comparisons of running activity between competitive matches and different types of various-sided games are necessary for coaches and practitioners in professional soccer [14], enabling the identification of specific drills that elicit similar, greater, or lower loads compared to actual match play [15]. Previous studies that compared the requirements of small-sided games and friendly matches have been carried out, but conclusive results were not obtained from them [15, 16]. Although Dellal et al. [16] found that soccer players achieved more high-intensity running and sprints in small-sided games, Casamichana et al. [17] asserted that greater amounts of sprinting were achieved in friendly matches. Studies comparing the demands of small-sided games with official matches are also scarce. Existing studies suggest that small-sided games can overemphasise acceleration and deceleration requirements while underestimating high-intensity running, compared to official matches for professional players [13, 14, 18–21].

Currently, many elements must be considered in the design of small-sided games to identify the constraints that better reproduce the match requirements [22]. One particularly innovative modification is the introduction of position games, which are small-sided games where soccer players must perform according to specific role positions, aiming to replicate similar situations encountered in competitive play [6]. Position games have not been well studied in the literature. To date, to the best of the authors’ knowledge, only one study analysing the external load of position games and traditional possession small-sided games has been published in the scientific literature [6], confirming that these drills have different running requirements, with greater demands in terms of accelerations and decelerations in the position games and greater distance covered, peak speed, and player load in the case of possession games. This study [6] also highlighted differences in the external load of position games of varying sizes, demonstrating that larger formats result in more distance covered, distance above 21 km · h^−1^, intense accelerations and decelerations, and player load. Meanwhile, smaller formats are associated with more accelerations and decelerations of lower intensity. Although position games are frequently used by soccer coaches in weekly training programmes, they have not been compared with official matches. For this reason, the objectives of this study were (1) to compare the external load of position games and official matches in professional youth soccer players and (2) to assess the effect of different pitch sizes in position games (Small, Medium, and Large) in relation to competition.

## MATERIALS AND METHODS

## Subjects

Twenty-five youth soccer players from the second team of a professional Spanish first division league participated in this study (age: 21.9 ± 1.9 years; height: 177.9 ± 5.2 cm; weight: 75.5 ± 4.8 kg; % body fat (Faulkner): 11.1 ± 1.4%). The study uniquely included data from players who completed entire matches, excluding those who were substituted or injured. Furthermore, data from goalkeepers were excluded. Data were collected from daily workload monitoring during team training sessions and official matches; therefore no ethics committee approval was required [23]. However, the study was in accordance with the recommendations of the Declaration of Helsinki, and the participants were informed of the study design and objectives, giving their consent before starting it.

## Procedures

Throughout the 2021–2022 season, a comparative analysis was performed using a descriptive design to assess the external load in position games and official matches. Using GPS systems to measure the players’ load, a total of 37 official matches and 18 training sessions were evaluated, resulting in 328 and 193 individual data points, respectively. All matches were part of the regular season, while training sessions were part of the daily monitoring of the squad. Both home and away matches were monitored, taking place on a field that adhered to the minimum standard size of 100 × 64 metres as established by the Fédération Internationale de Football Association. Therefore, the minimum area relative to each player was consistently > 320 m^2^, excluding goalkeepers. The predominant system used in matches was 1-4-3-3, although the 1-4-2-3-1 formation was also used.

**FIG. 1:**
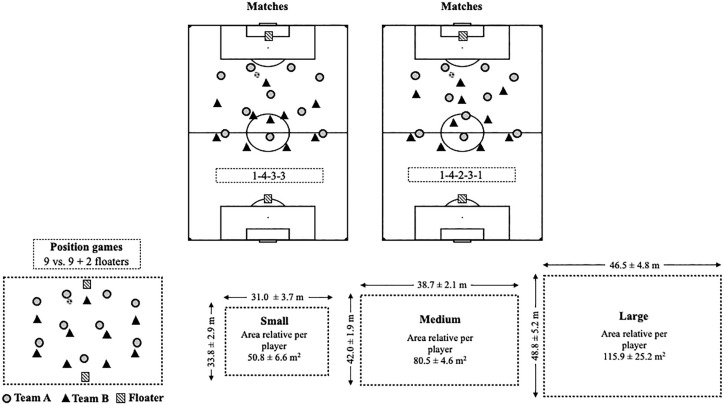
Graphical representation.

The three position games were conducted in a 9 vs. 9 + 2 floater players format, and categorised based on their pitch sizes: Small (33.8 ± 2.9 m × 31.0 ± 3.7 m, relative area per player = 50.8 ± 6.6 m^2^); Medium (42.0 ± 1.9 m × 38.7 ± 2.1 m, relative area per player = 80.5 ± 4.6 m^2^); and Large (48.8 ± 5.2 m × 46.5 ± 4.8 m, relative area per player = 115.9 ± 25.2 m^2^). During these games, soccer players were instructed to adhere to specific positional roles in a 4-2-3 system of play, a variation of 1-4-3-3 and 1-4-2-3-1, designed for 9 players on the team and 2 floating players (see Figure 1). The two floater players always participated with the team in the attacking role, providing support to the attacking team with the intention of facilitating ball possession, as the main objective of the task was to keep possession for as long as possible. Players were divided into two teams by the coach based on their positions and assessed skills, ensuring balanced teams. Additionally, two players acted as floaters, assuming roles in the goalkeeper or striker positions based on team possession, always playing with the offensive team. Each session began with a standardised 20-minute warm-up that included running, ball possession drills, and dynamic stretching exercises before progressing to designated drills. The tasks were executed in a continuous format lasting 8 minutes, allowing a maximum of two touches per player. These games were presented in a randomised sequence across different days within the 36-week regular season, aligning with the microcycle’s tactical and physical demands. The coaches actively engaged, providing verbal encouragement, constructive feedback and ensuring prompt ball reintroduction if the ball left the playing field.

To assess the external load on soccer players during matches and positional games, all players were equipped with GPS devices (WIMU Pro, RealTrack Systems, Almería, Spain) featuring a sampling rate of 10 Hz. The validity and reliability of this device in collecting timemotion variables have been thoroughly analysed and it has been established to be a suitable instrument for football purposes [24, 25]. Various metrics were recorded, including total distance covered (DC), distance covered above 21 km · h^−1^ (DC > 21 km · h^−1^), peak speed, maximal accelerations and decelerations, as well as accelerations lower and higher than 3 m · s^−2^ (Acc_<3_; Acc_>3_, respectively) and decelerations higher and lower than -3 m · s^−2^ (Dec_<3_; Dec_>3_). These specific variables have been used in prior studies [6]. To compare the values of intensity collected during position games and matches, all variables (except peak speed) were normalized by time.

## Statistical analysis

To confirm the data normality of each dataset, the Kolmogorov-Smirnov test, Q-Q plots of residuals, and random coefficient histograms were used. Data not following a normal distribution were transformed before further analysis [26]. Mixed model analyses were used to compare the effects of the game condition (i.e., Match, or position games) and the effects of space dimensions (Small, Medium, Large) of the position tasks and the competitive matches on the dependent parameters. The model used for each dependent parameter used game condition (i.e. Match, Games) and type of task (i.e. Small, Medium, Large, Match) as independent fixed factors and random intercepts on the individual player. A log-likelihood ratio test was used to assess the goodness of fit of the models. To assess the differences between each game condition, *post-hoc* analyses with Bonferroni’s correction were applied, while planned contrasts were specified to assess the differences between the size of each position game and the match condition, using *p*-values adjusted with Bonferroni’s correction. Standardized mean difference Cohen’s d effect sizes were obtained and were interpreted as: < 0.2 = trivial; 0.2–0.59 = small; 0.6–1. = moderate; 1.2–1.9 = large; > 2.0 = very large [27]. Statistical significance was set at α < 0.05. Unless otherwise stated, all values are presented as the estimated marginal mean (SE) or estimated marginal mean and 95% CI. The data analysis was performed using JAMOVI for Mac (version 2.3.13; The Jamovi project) and the Jamovi module GAMLj: General analyses for linear models [28].

## RESULTS

**FIG. 2:**
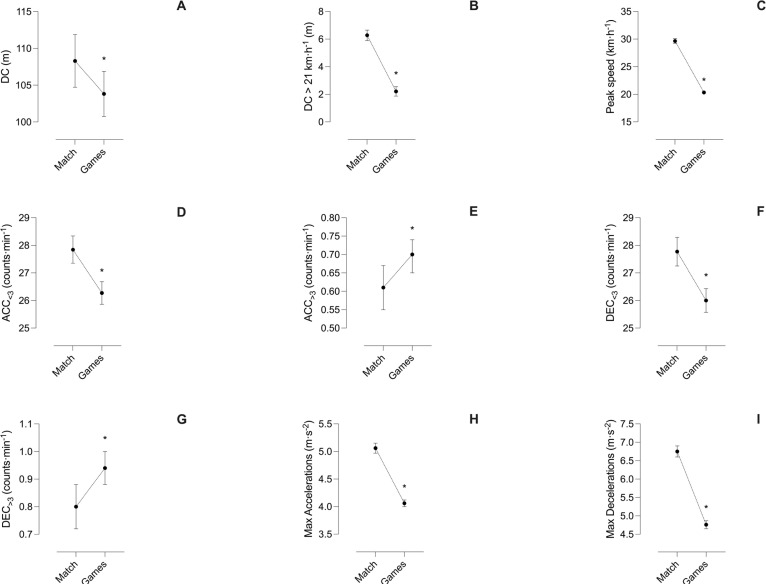
Comparative analyses between the matches and position games. A) DC, B) DC > 21 km · h^−1^, C) peak speed, D) ACC_<3_, E) ACC_>3,_ F) DEC_<3_, G) DEC_>3_, H) Max accelerations, G) Max decelerations. * = Statistically significant differences with Match condition. DC = distance covered; ACC = accelerations; DEC = decelerations; Max = maximal.

**FIG. 3:**
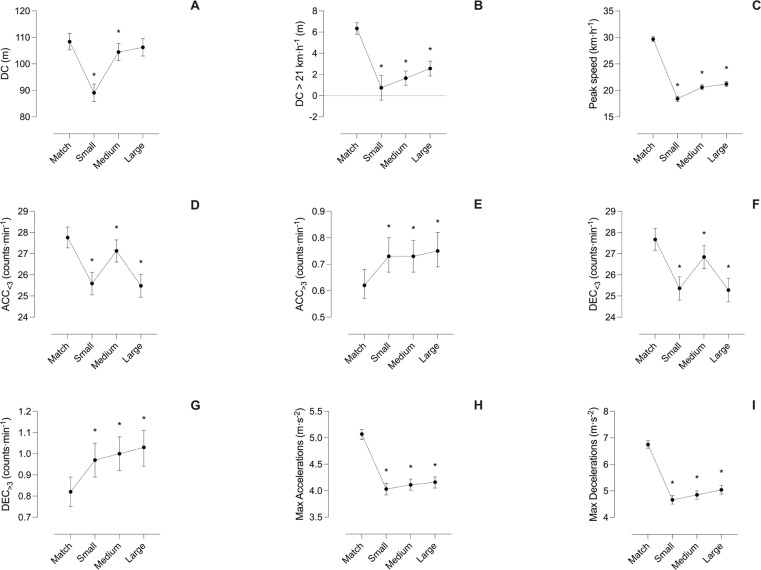
Comparative analyses between matches and Small, Medium and Large position games. A) DC, B) DC > 21 km · h^−1^, C) peak speed, D) ACC_<3_, E) ACC_>3,_ F) DEC_<3_, G) DEC_>3_, H) Max accelerations, G) Max decelerations. * = Statistically significant differences with Match condition. DC = distance covered; ACC = accelerations; DEC = decelerations; Max = maximal.

**FIG. 4:**
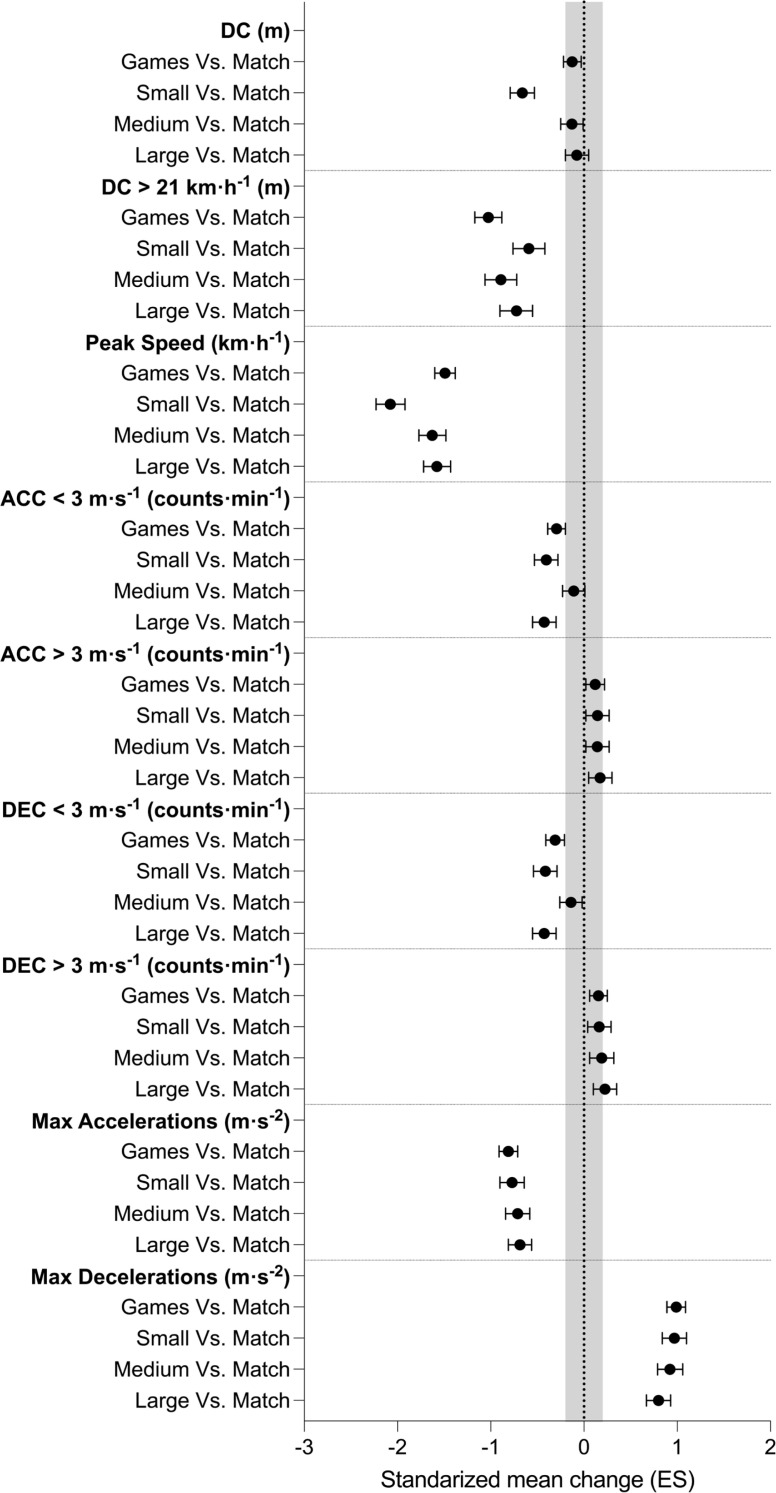
Effect sizes of differences between matches and Small, Medium and Large position games. * = Statistically significant differences with Match condition. DC = distance covered; ACC = accelerations; DEC = decelerations; Max = maximal. A value lower than zero denotes an effect size favouring the Match condition. The grey dashed vertical lines delimit the lower (-0.20) and upper limit (0.20) of a trivial effect size.

The external load values of the matches and position games are shown in Table 1, while the descriptive statistics and comparative analyses between them are illustrated in Figures 2, 3 and 4. All analysed variables reached statistical significance (all *p* < 0.001). During the position games, lower values of the variables DC (trivial), DC > 21 km · h^−1^ (moderate), peak speed (very large), Acc_<3_ (small), Dec_<3_ (small), maximal accelerations (moderate) and maximal decelerations (moderate) were observed than in competitive matches, with effect sizes ranging from trivial to very large. However, position games obtained higher values than matches for the Acc_>3_ and Dec_>3_ (trivial effect) variables.

**TABLE 1:** External load values of competitive Matches and Games.

Variable	Match	Games	*p* value	ES (95%CI)	Descriptor
DC (m)	106.55 ± 0.63	103.82 ± 1.49	< .001	-0.13 (-0.22–0.03)	Trivial
DC > 21 km · h^−1^(m)	6.48 ± 0.18	2.21 ± 0.17	< .001	-1.03 (-1.17–0.88)	Moderate
Peak speed (km · h^−1^)	30.23 ± 0.14	20.33 ± 0.13	< .001	-1.49 (-1.6–1.38)	Very large
ACC_<3_ (counts · min^−1^)	27.81 ± 0.11	26.27 ± 0.20	< .001	-0.29 (-0.39–0.20)	Small
ACC_>3_ (counts · min^−1^)	0.63 ± 0.01	0.70 ± 0.02	< .001	0.12 (0.02–0.22)	Trivial
DEC_<3_ (counts · min^−1^)	27.74 ± 0.11	26.00 ± 0.21	< .001	-0.31 (-0.41–0.21)	Small
DEC_>3_ (counts · min^−1^)	0.81 ± 0.01	0.94 ± 0.03	< .001	0.16 (0.06–0.25)	Trivial
Max ACC (m · s^−2^)	5.19 ± 0.04	4.06 ± 0.03	< .001	-0.81 (-0.91–0.71)	Moderate
Max DEC (m · s^−2^)	6.85 ± 0.05	4.76 ± 0.05	< .001	0.99 (0.89–1.09)	Moderate

Values are presented as mean ± SD. Match: mean values of the competitive matches; Games: mean values of the games; ES: Cohen’s d effect size; CI: Confidence interval; Descriptor: qualitative interpretation of the effect size. DC = Total distance covered; ACC = Accelerations; DEC = Decelerations; Max = Maximal.

The external load values of the matches and the conditions of the Small, Medium, and Large position games are represented in Table 2 and the descriptive statistics and comparative analyses are shown in Figures 3 and 4.

**TABLE 2:** External load values of competitive matches and different sizes of position games.

Variable	Match	Small	Medium	Large
DC (m)	106.55 ± 0.63	89.03 ± 1.76* ^S^	104.47 ± 1.71* ^T^	106.25 ± 1.74
DC > 21km · h^−1^(m)	6.48 ± 0.18	0.74 ± 0.59* ^S^	1.65 ± 0.34* ^M^	2.56 ± 0.36* ^M^
Peak speed (km · h^−1^)	30.23 ± 0.14	18.41 ± 0.24* ^VL^	20.58 ± 0.24* ^VL^	21.20 ± 0.24* ^VL^
ACC_<3_(counts · min^−1^)	27.81 ± 0.11	25.59 ± 0.27* ^S^	27.13 ± 0.26* ^T^	25.48 ± 0.27* ^S^
ACC_>3_(counts · min^−1^)	0.63 ± 0.01	0.73 ± 0.03* ^T^	0.73 ± 0.03* ^T^	0.75 ± 0.03* ^T^
DEC_<3_(counts · min^−1^)	27.74 ± 0.11	25.36 ± 0.28* ^S^	26.84 ± 0.27* ^T^	25.28 ± 0.28* ^S^
DEC_>3_(counts · min^−1^)	0.81 ± 0.01	0.97 ± 0.04* ^T^	1.00 ± 0.04* ^T^	1.03 ± 0.04* ^S^
Max ACC (m · s^−2^)	5.19 ± 0.04	4.03 ± 0.05* ^M^	4.11 ± 0.05* ^M^	4.16 ± 0.05* ^M^
Max DEC (m · s^−2^)	6.85 ± 0.05	4.66 ± 0.08* ^M^	4.85 ± 0.08* ^M^	5.04 ± 0.08* ^M^

Values are presented as mean ± SD. Match: mean values of competitive matches; Small: mean values of small position games; Medium: mean values of medium position games; Large: mean values of large position games;

* *p* ≤ 0.05 statistically significant from Match values.

T trivial effect size;

S small effect size;

M moderate effect size;

VL very large effect size; DC = Total distance covered; ACC = Accelerations; DEC = Decelerations; Max = Maximal.

In Small and Medium position games, all variables analysed showed statistically significant differences (all *p* < 0.05). The comparison between the Large position games and the matches showed significant differences in all variables (all *p* < 0.05) except for DC (*p* = 0.094). Regarding Small position games, lower values were achieved than in matches for DC (small), DC > 21 km · h^−1^ (small), peak speed (very large), Acc_<3_ (small), Dec_<3_ (small), maximal accelerations (moderate), maximal decelerations (moderate). However, significantly higher values were reported than in the matches for Acc_>3_ (trivial) and Dec_>3_ (trivial). In the case of the Medium position games, lower values than in the matches were registered for DC (trivial), DC > 21 km · h^−1^ (moderate), peak speed (very large), Acc_<3_ (trivial), Dec_<3_ (trivial), maximal accelerations (moderate) and maximal decelerations (moderate). However, higher values than in the matches were reported for Acc_>3_ (trivial) and Dec_>3_ (trivial).

Finally, in the case of the Large position games, lower values than in the matches were recorded for DC > 21 km · h^−1^ (moderate), peak speed (very large), Acc_<3_ (small), Dec_<3_ (small), maximal accelerations (moderate) and maximal decelerations (moderate). However, higher values than in the matches were achieved for Acc_>3_ (trivial), and Dec_>3_ (small).

## DISCUSSION

The aims of this study were to compare the external load of positional games and official matches in youth professional soccer players and to assess the effect of different pitch sizes in positional games (Small, Medium, and Large) in relation to competition. The main finding of this study was that during matches, soccer players achieved more DC > 21 km · h^−1^, peak speed, Acc_<3_, Dec_<3_, maximal accelerations and maximal decelerations compared to position games, regardless of their size. However, in all three formats analysed, players achieved more Acc_>3_ and Dec_>3_ than in the matches.

The comparison between the requirements of matches and the demands of training sessions is a crucial aspect of soccer training [14]. Despite its importance, there is a paucity of research that systematically compares training tasks with real match scenarios [18, 29]. This is the first study to compare the external load of official games and position games. Our results indicate that matches and position games have different running requirements. These results are consistent with previous published literature, which has also found differences between matches and small-sided games [14, 16–18, 20, 30, 31], as well as matches and other drills such as transition games [32]. In this study, soccer players achieved higher DC, DC > 21 km · h^−1^ and peak speed during matches compared to position games. The higher demands of the matches could be attributed to the differences in the format used during the position games (9 vs. 9 + 2 compared to 11 vs. 11 in matches) and to the greater relative area per player in competition (> 320 m^2^) compared to training tasks, where the larger formats had an average relative area per player of 115.9 m^2^. Currently, it is also known that as the number of players and pitch size increase in small-sided games, the demands of these variables tend to increase [14]. Although a previous study in this field found higher demands for DC, high-intensity running, and sprint distance during small-sided games compared to matches [16], most published studies seem to agree that competition tends to be more demanding than small-sided games in terms of distance covered at high speed, sprints and peak speed, and lower in relation to DC [13, 14, 17, 18]. However, it is true that these results can be modified depending on the format of the smallsided games used, as factors such as the skill levels of the players or other possible constraint factors (e.g., number of players, number of touches, duration of execution, orientation of the field, or playing rules) could yield different results [14, 33, 34]. This could explain, for example, why some studies have found differences in terms of high speeds between smaller-sided games (e.g., 5 vs. 5 and 6 vs. 6) and matches, and these differences are reduced when larger formats are used (e.g., 10 vs. 10) [20].

Regarding variables related to speed changes, official matches exhibited higher demands for ACC_<3_, DEC_<3_, maximal accelerations and maximal decelerations, while position games showed higher requirements for ACC_>3_ and DEC_>3_. In a recently published study comparing position games and small-sided games, it was found that the presence of a tactical rule, which required players to return to their original position after each movement, caused more frequent and more intense accelerations and decelerations during the positional games [6]. In the present study, two activities with imminent tactical characteristics were compared, matches and position games, with some aspects potentially explaining their differences. During matches, a higher percentage of time is spent at speeds in the range 0.0–6.9 km · h^−1^ compared to small-sided games [17], which could result in higher demands for low-intensity accelerations and decelerations, as well as maximum accelerations and decelerations during competition, since more space is needed to execute these actions [35]. On the other hand, in position games, due to the limited space available and players spending more time in close proximity to the ball, players may have been compelled to perform more frequent high-intensity accelerations and decelerations to fulfil their assigned tactical roles.

In an effort to gain a deeper understanding of position games, this study also compared differentiated position games based on their size with actual matches. The results were consistent with those mentioned above, without differentiating the size of the position games, with the sole exception that there were no differences in DC between the Large position games and the matches (see Figure 3). These results are in line with what has been previously stated in the literature, where it appears that small-sided games performed in large spaces make it possible to achieve the highest similarity with competition for kinematic and mechanical variables. However, when played in smaller spaces, they could induce an overload in mechanical parameters [31]. The identified differences may be linked to variations in the relative area per player across Small, Medium, and Large position games and matches. Previous research has highlighted the importance of the relative area per player as a determinant of training workload [35–37]. For example, a systematic review and meta-analysis examining small-sided games revealed that a difference of 25 m^2^ in the relative area per player could induce shifts in the demands placed on soccer players in terms of highspeed running, sprinting, and other related parameters [36]. Considering that the relative area per player differed by 270 m^2^, 240 m^2^, and 205 m^2^ in Small, Medium, and Large position games, respectively, this factor likely played a significant role. The reduced size of the pitches in position games would result in shorter distances between players [38], requiring players to cover less ground (implying lower DC, Acc_<3_, Dec_<3_). Simultaneously, the spatial limitations inherent in position games would restrict the occurrence of high-intensity actions that require more space (DC > 21 km · h^−1^ and peak speed), while promoting the accumulation of greater Acc_>3_ and Dec_>3_, albeit not reaching the levels observed in full matches in terms of maximal accelerations and maximal decelerations.

A previous study asserted that position games, similar to matches, entail distinct external loads compared to small-sided games when reproduced under identical characteristics [6]. Despite these differences, position games, such as small-sided games, are often chosen by coaches to meet game demands because of their ability to simultaneously develop multiple aspects of performance [39]. This study, along with previous studies conducted with small-sided games [20, 36], highlights that these types of tasks rarely replicate all the demands of the match. Coaches should understand that different game formats could lead to different load stimuli [31]. For this reason, it is recommended to use position games in combination with other types of tasks, such as transition games or small-sided games [14, 36, 37, 40, 41], to achieve optimal development of football players for competition.

Although this study provided novel findings about the application of position games in soccer, some limitations must be acknowledged when interpreting these results. The main limitation of this study is that it investigates only one game format, and therefore the results are limited to a specific condition. Caution should be exercised when extrapolating this knowledge to other types of training tasks or game formats. For example, in this investigation, the largest position games had a mean individual interaction area of 115.9 m^2^. Considering that the individual interaction area in an actual match is substantially larger, future studies should explore position games with expanded areas. Likewise, the studied format only involved 9 players per team and 2 wildcards. Given that real games lack wildcards and typically involve two teams of 11 players each, it is important to investigate the physiological demands of position games under such conditions in future research. Lastly, this study solely analysed the external load of players, without assessing potential differences in internal load between position games and actual matches. Therefore, future investigations should investigate the internal load imposed by position games in football.

## CONCLUSIONS

This article presents novel insights into football training that can be applied to daily training and help better understand the tasks commonly used by coaches, such as position games. The data reported in this work showed that position games with a format of 9 vs. 9 + 2 and a relative area per player ≤ 115.9 m^2^ presented a different external load compared to official matches. Coaches could use the position games described in this study in training sessions aimed at stimulating high-intensity accelerations and decelerations, for example, during the conditioning phase of the training microcycle. Similarly, if the training objective is to improve a player’s ability to perform actions at high speed or maximal accelerations and decelerations, these position games could be used but should be complemented with other tasks as they have lower requirements in these variables compared to matches. Considering the lower demands compared to matches, it would also be possible to use these games in the final phase of the microcycle (days before the match), as they would not cause excessive fatigue, allowing players to perform according to specific role positions, to replicate similar situations encountered in competitive play.
